# Supplementary material for: Liposomal Rifabutin—A Promising Antibiotic Repurposing Strategy against Methicillin-Resistant Staphylococcus aureus Infections
Source: Pharmaceuticals (Basel). 2024 Apr 8;17(4):470. doi: 10.3390/ph17040470 (PMC11053623; doi:10.3390/ph17040470)
Supplement: Supplementary file 1 [file pharmaceuticals-17-00470-s001.zip › pharmaceuticals-2898137-supplementary.pdf]

Supplementary Materials

Liposomal Rifabutin—A Promising Antibiotic Repurposing Strategy against Methicillin-Resistant Staphylococcus Aureus Infections

Jacinta O. Pinho [1], Magda Ferreira [1,2,3], Mariana Coelho [1], Sandra N. Pinto [4], Sandra I. Aguiar [2] and Maria Manuela Gaspar [1,5,*]

1 Research Institute for Medicines (iMed.ULisboa), Faculty of Pharmacy, Universidade de Lisboa, Av. Prof. Gama Pinto, 1649-003 Lisboa, Portugal; pinho.jacinta@campus.ul.pt (J.O.P.); magda.ferreira@campus.ul.pt (M.F.); mariana.coelho@ff.ulisboa.pt (M.C.)
2 Center for Interdisciplinary Research in Animal Health (CIISA), Faculty of Veterinary Medicine, Universidade de Lisboa, 1300-477 Lisboa, Portugal; siraguiar@gmail.com
3 Associate Laboratory for Animal and Veterinary Sciences (AL4AnimalS), Faculty of Veterinary Medicine, Universidade de Lisboa, 1300-477 Lisboa, Portugal
4 iBB-Institute for Bioengineering and Biosciences and Associate Laboratory i4HB−Institute for Health and Bioeconomy at Department of Bioengineering, Instituto SuperiorTécnico, Universidade de Lisboa,
1049-001 Lisboa, Portugal; sandrapinto@ist.utl.pt
5 IBEB, Institute of Biophysics and Biomedical Engineering, Faculty of Sciences, Universidade de Lisboa, Campo Grande, 1749-016 Lisboa, Portugal
* Correspondence: mgaspar@ff.ulisboa.pt

Supplementary Methods

a) Stability of RFB-loaded liposomes in suspension

The stability of RFB liposomal suspensions, DMPC:DMPG:DSPE-PEG (65:30:5; RFB-LIP1) and DPPC:DPPG:DSPE-PEG (65:30:5; RFB-LIP2), was assessed after storage at 4 °C during 7. Briefly, RFB liposomes were prepared as described in Section 3.3. RFB-LIP1 and RFB-LIP2, suspended in HEPES buffer pH 6.9 (10 mM HEPES, 140 mM NaCl), were stored at 4 °C during 7 days. At tested time, RFB liposomes were placed in a desalting column (Econo-Pac® 10 DG; Bio-Rad Laboratories, Hercules, CA, USA) to remove the released antibiotic. After, liposomal suspensions were ultracentrifuged at 250,000*g*, for 120 min, at 15 °C, in a Beckman LM-80 ultracentrifuge (Beckman Instruments, Inc, Fullerton, CA, USA). Obtained pellets were suspended in HEPES buffer, pH 6.9, and RFB and phospholipid contents were determined. The stability of RFB-LIP1 and RFB-LIP2 was defined as the ratio, in percentage, between RFB to lipid ratio at analyzed time (7 days) and the RFB to lipid ratio at time = 0 days (equation S1). Mean hydrodynamic size and PdI of samples were also evaluated.

$$\%\text{ of RFB associated within liposomes} = \frac{\left(\frac{\text{RFB}}{\text{Lipid}}\right)_{7/\text{days}}}{\left(\frac{\text{RFB}}{\text{Lipid}}\right)_{0\text{ days}}} \times 100 \qquad \text{(Equation S1)}$$

Supplementary Figures

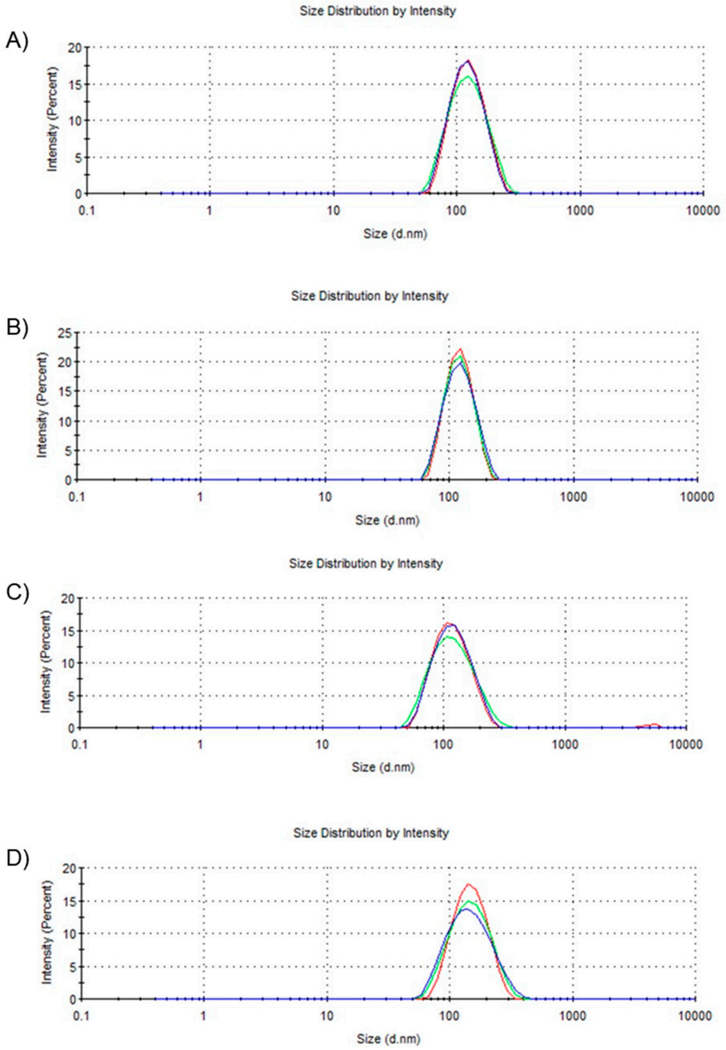

Figure S1. Liposomes size distribution. A) RFB-loaded DMPC:DMPG:DSPE-PEG (RFB-LIP1); B) Unloaded DMPC:DMPG:DSPE-PEG (Unloaded-LIP1); C) RFB-loaded DPPC:DPPG:DSPE-PEG (RFB-LIP2); D) Unloaded DPPC:DPPG:DSPE-PEG (Unloaded-LIP2).

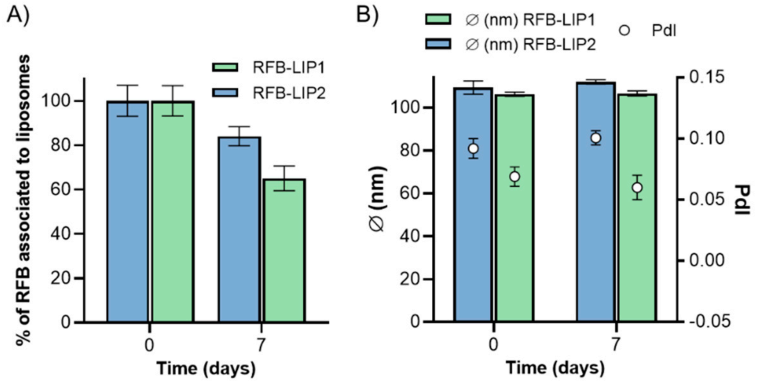

Figure S2. Stability of RFB-loaded liposomes, RFB-LIP1 and RFB-LIP2, after storage in buffer at 4 °C, for 7 days. A) Percentage of RFB associated within liposomes and B) Mean hydrodynamic size and PdI. RFB-LIP1:

DMPC:DMPG:DSPE-PEG; RFB-LIP2: DPPC:DPPG:DSPE-PEG; RFB: rifabutin; PdI: polydispersity index. Results are expressed as mean ± SD of three independent experiments.

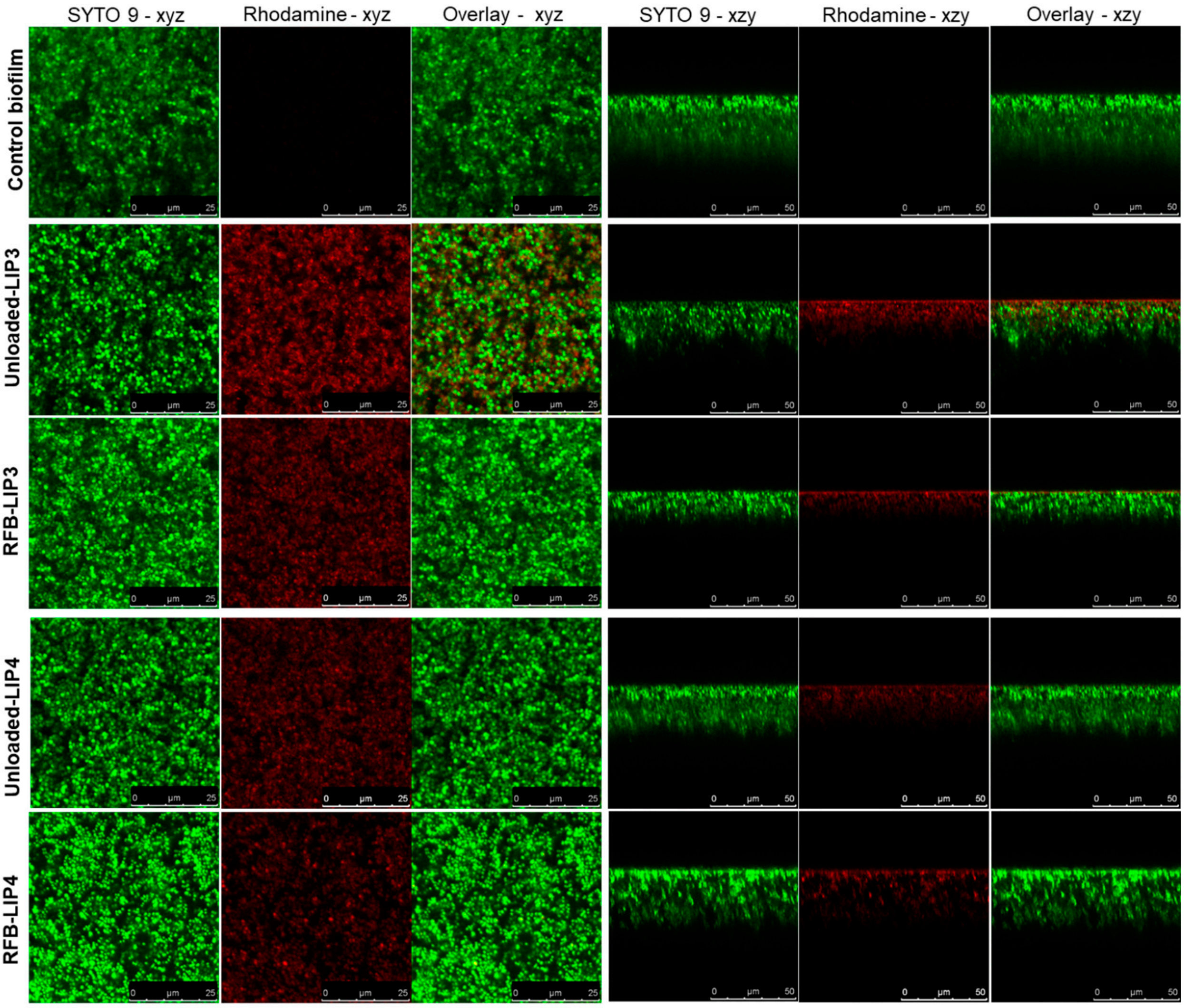

Figure S3. Representative CSLM images of 24 h-old MRSA-C1 biofilms after incubation with rhodamine-labelled LIP3 and LIP4 liposomes, unloaded and RFB-loaded, at a lipid concentration of 1.5 μmol/mL, for 24 h. Biofilms were stained with the green dye SYTO 9 at 3 μM. Untreated biofilm was used as a control (Control biofilm). Images in the left panels correspond to xyz view, and images in the right panels correspond to xzy orthogonal view. The overlay of the green and red channels from each plane image is presented as Overlay - xyz and Overlay - xzy. Lipid compositions: DMPC:DMPG (RFB-LIP3 and Unloaded-LIP3); DPPC:DPPG (RFB-LIP4 and Unloaded-LIP4).

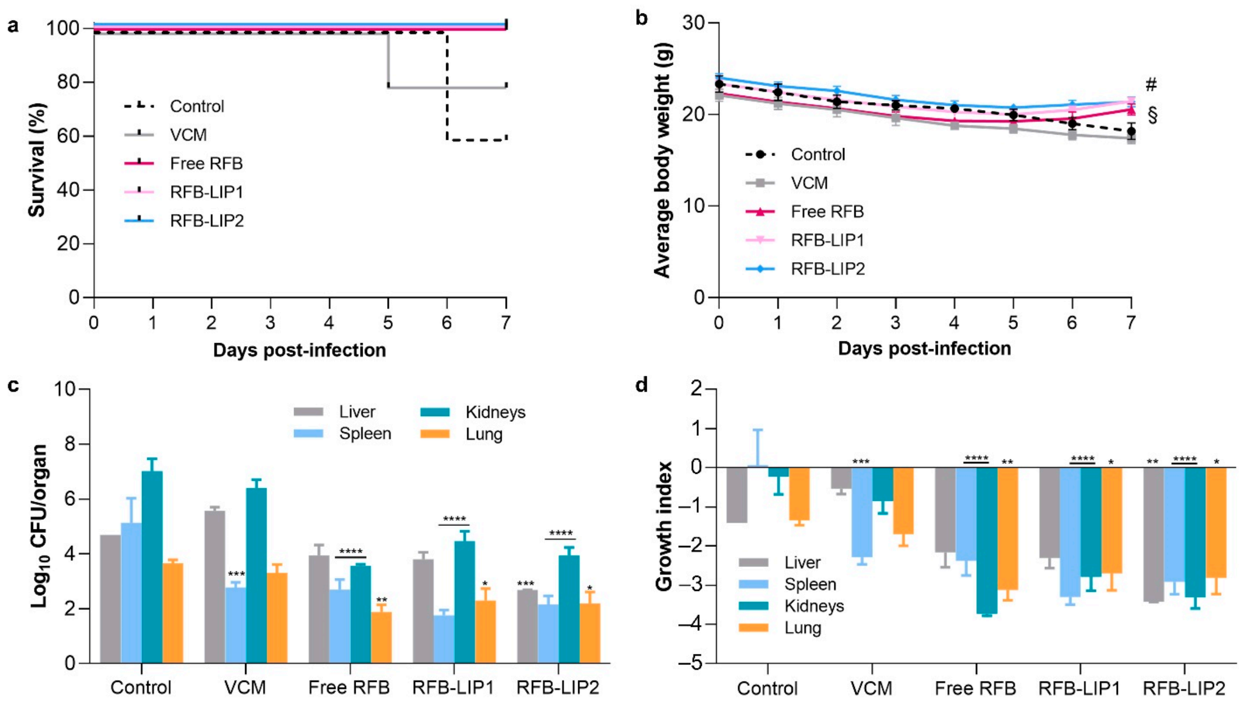

Figure S4. In Vivo Assay 2 – Therapeutic evaluation of RFB and VCM formulations in a murine model of systemic MRSA-C1 infection. Infection was induced intravenously in male Balb/c mice with a MRSA-C1 inoculum at $3.4{\times}10^8$ CFU/mouse. Three days after infection induction mice received IV administrations of VCM (40 mg/kg) and RFB formulations (Free RFB, RFB-LIP1 and RFB-LIP2; 20 mg/kg). Control group received buffer by intravenous route. (**a**) percentage of survival (Kaplan-Meier analysis), (**b**) average body weight, (**c**) bacterial burden in major organs at the end of treatment protocol, and (**d**) growth index. Lipid compositions: DMPC:DMPG:DSPE-PEG (RFB-LIP1) and DPPC:DPPG:DSPE-PEG (RFB-LIP2). Results are expressed as mean ± SEM (n=5-6). Two-way ANOVA with Dunnett's test. #$p<0.05$ (Free RFB vs Control) and § $p<0.01$ (RFB-LIP1 and RFB-LIP2 vs Control) at day 7 post-infection; *$p<0.05$, **$p<0.01$, ***$p<0.001$, ****$p<0.0001$ vs Control group.

Table S1. Tissue indexes of liver, spleen, kidneys, and lung of healthy mice (naïve) and mice infected with systemic MRSA-C1 and treated with tested formulations. In Vivo Assay 1 corresponds to mice induced with 1.4×10^{9} CFU/mouse and In Vivo Assay 2 to animals induced with 3.4×10^{8} CFU/mouse. Results are expressed as AVG ± SEM (n=4-5).

In vivo assay	Group	Tissue Index (AVG ± SEM)			
		Liver	Spleen	Kidneys	Lung
**In Vivo Assay 1** (1.4×10^{9} CFU/mouse)	**Control** (beginning of treatment)	25.6 ± 0.1	7.9 ± 0.3	15.1 ± 1.0	8.8 ± 0.2
	**Control**	25.6 ± 0.6	8.4 ± 1.0	15.5 ± 0.2	9.9 ± 0.5
	**Free RFB**	23.2 ± 0.3	6.9 ± 0.5	13.6 ± 0.5	9.8 ± 0.3
	**RFB-LIP2**	23.9 ± 0.7	8.8 ± 0.5	14.0 ± 0.5	9.2 ± 0.2
**In Vivo Assay 2** (3.4×10^{8} CFU/mouse)	**Control** (beginning of treatment)	25.3 ± 0.4	8.0 ± 0.1	12.5 ± 0.3	9.8 ± 0.3
	**Control**	25.4 ± 0.4	10.2 ± 0.2	14.4 ± 0.1	10.4 ± 0.4
	**VCM**	25.1 ± 0.6	8.4 ± 0.4	14.0 ± 0.3	9.6 ± 0.3
	**Free RFB**	24.9 ± 0.4	7.2 ± 0.2	13.1 ± 0.2	9.1 ± 0.2
	**RFB-LIP1**	26.1 ± 0.4	7.8 ± 0.3	13.4 ± 0.1	9.0 ± 0.3
	**RFB-LIP2**	25.0 ± 0.5	8.0 ± 0.2	13.3 ± 0.2	8.8 ± 0.4
N.A.	**Naïve**	24.0 ± 0.4	5.9 ± 0.3	13.0 ± 0.2	7.2 ± 0.3

RFB: rifabutin; VCM: vancomycin; CFU: colony forming units; Lipid compositions: DMPC:DMPG:DSPE-PEG (RFB-LIP1) and DPPC:DPPG:DSPE-PEG (RFB-LIP2); N.A.: not applicable.

Table S2. In Vivo Assay 2 – Score of analysed organs in terms of inflammation/necrosis (liver, kidney, and lung) and reaction to infection (spleen). Systemic infection was induced with 3.4×10^{8} CFU/mouse.

	Inflammation/Necrosis			Reaction to infection
	Liver	Kidney	Lung	Spleen
**Control**	1	4	0	1
**VCM**	0	3	0	1
**Free RFB**	0	0	0	2
**RFB-LIP1**	0	0	0	1
**RFB-LIP2**	0	1	0	1

Inflammation/necrosis score: 0 = within normal limits; 1 = single cell necrosis; 2 = small foci of necrosis/abscesses; 3 = multifocal large foci of necrosis/abscesses; 4 = extended coalescing foci of necrosis/abscesses. Spleen reaction to infection: 0 = within normal limits; 1 = minimal; 2 = mild; 3 = moderate; 4 = severe.
